# Patient journeys for neglected tropical diseases in rural sub-Saharan Africa: a scoping review

**DOI:** 10.1186/s40249-025-01385-7

**Published:** 2025-11-06

**Authors:** Sandrena Ruth Frischer, Eloise Ockenden, Fabian Reitzug, Michael Parker, Goylette F. Chami

**Affiliations:** 1 Big Data Institute, Nuffield Department of Population Health, University of Oxford, Oxford, UK; 2 Ethox Centre, Nuffield Department of Population Health, University of Oxford, Oxford, UK

**Keywords:** Neglected tropical diseases, Patient journey, Continuity of care, Care seeking, Illness narrative, Referral, Sub-Saharan Africa

## Abstract

**Background:**

Patient journeys highlight evolving processes of care seeking from patient perspectives over the course of time and disease progression. Patient journeys for neglected tropical diseases (NTDs) in rural sub-Saharan Africa (SSA) are poorly understood. This review aims to identify studies including patient journeys for NTDs in rural SSA.

**Methods:**

Systematic search of six scientific databases from inception to 18 November 2024**.** All studies were required to include patient journeys for NTDs, defined as the continuous arc of the patient care seeking experience at multiple time points while navigating increasingly debilitating disease. All patient journeys were depicted explicitly using flow diagrams, lists of ordered journey components, or patient narratives. Variables extracted included the use and rationale of referrals, types of healthcare delivery providers engaged in the patient journey, and barriers and facilitators of care continuity. Journeys were analysed using framework synthesis.

**Results:**

Searches returned 2605 studies where after de-duplication and eligibility screening, 22 studies were identified for inclusion Included studies represented eight NTDs, which were categorised into four groups: severe and stigmatising skin NTDs (SSSDs) (13/22) including Buruli ulcer, lymphatic filariasis, onchocerciasis, and yaws; human African trypanosomiasis (HAT) (3/22); snakebite and rabies (4/22); and schistosomiasis (intestinal and female genital) (2/22). NTD patient journeys revealed health system constraints relating to limited medical resources and ineffective referral pathways, social dimensions of gender and stigma hindering access to care, and logistical concerns related to distance to health facilities, and lack of transport. Patient journeys for different NTDs highlighted specific dimensions of this local context, including challenges with mental health distress for individuals living with SSSDs, difficulties obtaining diagnoses for HAT as an NTD with non-specific symptoms, and inaccessibility of treatment for schistosomiasis in the context of missed mass drug administration.

**Conclusions:**

NTD patient journeys show varied care seeking experiences within the broader context of neglect and health inequity that characterises settings where NTDs are endemic. For NTDs resulting in long-term or chronic conditions, these journeys highlight inaccessible care and a lack of integrated approaches for prevention, treatment, and management within health systems. By understanding patient journeys, NTD researchers and practitioners can determine how best to support NTD patients in navigating access to care.

**Graphical abstract:**

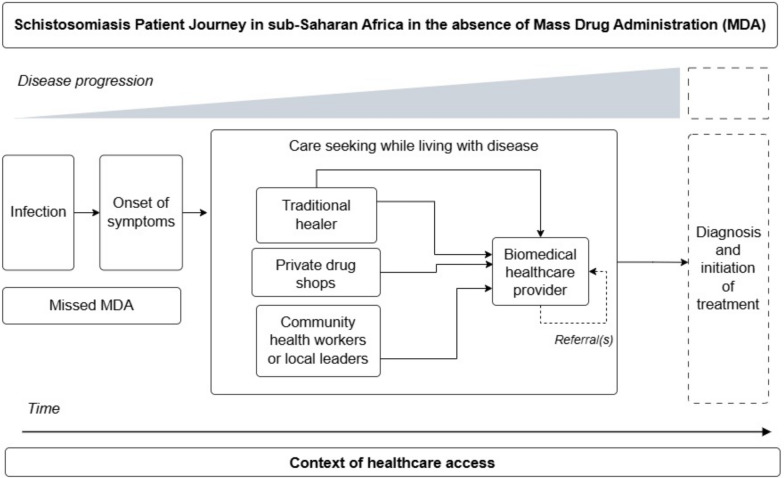

**Supplementary Information:**

The online version contains supplementary material available at 10.1186/s40249-025-01385-7.

## Background

Neglected tropical diseases (NTDs) comprise more than 20 diverse diseases that share the challenges of political deprioritisation and endemicity in settings of extreme poverty [1]. NTDs are prevalent in rural settings of many low and middle-income countries (LMICs), with the highest NTD burden globally in sub-Saharan Africa (SSA) [2]. Patient journeys and related frameworks, such as care pathways and patient pathways, have proven useful for understanding the routes individuals may take when attempting to access care for NTDs in resource-constrained environments in SSA [3–6]. Patient journeys illuminate patient perspectives navigating accessing to care, typically through detailed patient narratives or schematics which emphasize patient experiences of illness and agency in care seeking preferences [7]. Through emphasis on evolving care needs over the course of time and disease progression, patient journeys go beyond identification of fixed barriers and facilitators to care to highlight patient perspectives when transitioning from care seeking and diagnosis to treatment and follow up [8]. Patient journeys are thus well suited for understanding processes of healthcare seeking for NTDs in LMIC settings where health systems infrastructure may be limited and recommended care for complex conditions requiring integrated case management may not be available [9].

Using a pathway framework to conceptualise barriers and facilitators of care for severe and stigmatising skin NTDs (SSSDs), a recent scoping review by McCollum and colleagues was conducted in LMICs [10]. SSSDs include Buruli ulcer, leprosy, onchocerciasis, lymphatic filariasis, and yaws. This review provides a rich picture of the complexity faced by SSSD patients attempting to access care, highlighting supernatural beliefs and stigma as highly influential aspects of patient experiences. Given the SSSD scope, the barriers and facilitators of care identified in the McCollum review do not necessarily extend to other non-SSSD NTDs with different manifestations or management strategies, such as human African trypanosomiasis (HAT) [11, 12], schistosomiasis [13], snakebite or rabies [14]. However, non-SSSD NTDs are endemic in the same settings as SSSDs, are similarly associated with neglect and poverty, and are also associated with the development of disability [15]. Additionally, for both SSSDs and many NTDs more generally, symptoms are initially non-specific and may only become evident upon progression to severe disease, suggesting similar challenges relating to disease identification and diagnosis [2]. Recognizing the similarities and differences between NTDs and their varying care needs, there is a need to understand evolving NTD care seeking experiences over time for all NTDs. The scoping review reported here uses a patient journey framework to understand the experiences of individuals accessing care for all NTDs in rural SSA, and explore how these journeys are represented in the published literature. This review builds on the McCollum review by conceptualising the experiences of care seeking across all NTDs over the course of time and disease progression. In this review, we address the following questions: What are patient journeys for NTDs in SSA? How are patient journeys similar or different between NTDs?

## Methods

This review followed the Joanna Briggs Institute methodological guidance for conducting systematic scoping reviews, with reporting in line with the 2018 Preferred Reporting Items for Systematic Reviews and Meta Analyses extension for Scoping Reviews (PRISMA-ScR) (S1 Text) [16, 17]. This review is the result of a sub-analysis from a larger review, the protocol for which was published on 28 February 2023 on the Open Science Framework [18].

### Search strategy

Guided by a 2019 scoping review on patient pathways within integrated healthcare [9], two researchers (SRF and GFC) developed the search string through discussion and iterative search piloting using terminology relating to patient journeys. Given similarity to patient care concepts such as the “care continuum” and “treatment cascade”, we opted to include all terminology identified as potentially synonymous to patient journeys or relating to patient experiences accessing care over time. We also elected to include “referrals” in the search string to explicitly incorporate how patients move between journey components, such as from suspect case assessment or diagnosis to follow up. All NTDs as recognized by the World Health Organisation (WHO) were included in the search string [19]. This inclusion of all NTDs allows for comparison of similarities and differences of pathways between NTDs. We restricted geographic scope to countries in SSA as categorised by the World Bank to focus on healthcare delivery contexts specific to the region with the highest NTD burden. The broad search string included “patient pathways” AND “neglected tropical diseases” AND “SSA”. Using the broad string with adaptations applied as necessary, one researcher (FR) searched the following databases on 16 February 2023 without year restrictions: PubMed/MEDLINE, Embase, Global Health, Global Index Medicus, and Web of Science Core Collection—Science Citation Index Expanded. The same researcher (FR) also searched the Cochrane Central Register of Controlled Trials (established 1996-present) for randomised controlled trials before uploading all resulting articles into Covidence (Veritas Health Innovation, Melbourne, Australia) for deduplication and eligibility screening. The full string and table of database hits is available in S2 Text. The full search was rerun on 18 November 2024 by SRF. Following both runs of the search, we complemented the search with citation searching of included studies within the same timeframe as the original searches. This included backward citation searching of all citations from the included studies, and forward citation searching using Google Scholar to identify which studies had cited the original included studies.

### Screening and concept development

Two researchers (SRF and EO) screened all titles and abstracts resulting from the search in duplicate. The full text of all eligible studies was then retrieved, where available. All full texts were screened by one researcher (SRF), with a random 10% verified by a second researcher (EO). Disagreements between researchers were resolved through discussion and review of the full text. Where applicable, studies describing the same population at the same time point were selected based on the study providing the most information. To centre NTD patient experiences, all studies were required to include either current, likely suspect, or former NTD patients in the sample population. By requiring studies engage NTD patients and use of qualitative methods such as interviews, observation, and focus groups, we aimed to ensure NTD patient journeys focused on patient experiences and perceptions of those experiences throughout the process of care seeking care. We elected to exclude grey literature and unpublished literature, such as conference abstracts and doctoral theses, to provide a clear representation of how patient journeys have been represented for NTDs in published research. Studies limited to clinical case reports were excluded given case report focus on atypical case presentation.

Only studies including patient journeys for NTDs met criteria for inclusion. However, the concept of patient journeys relates to and intersects with similar concepts relating to patient experiences accessing healthcare, such as healthcare seeking pathways, care pathways, and treatment algorithms. These concepts are often used interchangeably and without differentiation. To distinguish patient journeys from these similar concepts within the context of NTDs and in the scope of this review, we developed a working definition of patient journeys for NTDs through discussion and review of the literature (Table 1). As more clinically advanced cases may require more complex care, the continuous arc of the patient journey contrasts with an experience of care seeking based on barriers and facilitators to care at a singular point in time, irrespective of how care needs may change in line with disease progression. A summary of all inclusion and exclusion criteria is presented in Table 1.

**Table 1:** Summary table of inclusion and exclusion criteria

Inclusion criteria	Exclusion criteria
Original published research articles with primary data collection	Study designs not involving primary data collection, including all reviews (narrative, systematic, scoping)
Inclusion of current, former, or suspect patients in the study population	Unpublished literature, including grey literature
In English or with a published translation	Clinical case studies or trial protocols
Study setting in rural SSA	Studies limited to describing algorithms, cascades, or pathways of predefined recommended sequences of events
Employ qualitative methods, such as interviews, observation, and focus group discussions	Data from outside SSA, including of SSA nationals seeking care outside of SSA
Must include patient journeys for NTDs, defined as the continuous arc of the patient care seeking experience from the patient perspective. These patient journeys must: • Be depicted through either a flowchart, diagram, list, or narrative • Describe multiple time points, shown through either: explicit discussion of disease progression, reference to multiple attempts seeking treatment, or exploration of an individual’s experience of disease over a defined time period	

As part of this concept development, we recognized several distinctions between patient journeys and diagnostic or treatment algorithms or cascades, and care pathways. Studies describing diagnostic algorithms, treatment cascades, or care pathways typically involved a sequence of recommended steps to arrive at a confirmatory diagnosis. Comparatively, patient journeys were patient-directed and showcased actual patient experiences along the care continuum. These experiences typically deviated from recommended steps in algorithms or pathways, and often included steps outside of formal health systems, such as through engagement with traditional healers. Additionally, while we began with patient pathways as the primary terminology, during this concept development we shifted to patient journeys as a more representative term for describing the progressive, patient-directed arc of care seeking over time.

### Data extraction and analysis

Prior to data extraction, two researchers (SRF and GFC) developed an initial data dictionary and table for extracted variables using Microsoft Excel (Version 2108, Microsoft Corporation, Redmond, Washington, USA). We then split data extraction into three stages. In the first stage, we extracted study design and aims, country, NTD(s) of focus, and study population using the initial table. In the second stage we identified how patient journeys were depicted and themes common to these journeys. In this second stage, SRF revised, piloted and iterated the data dictionary and extraction table to capture depictions and themes of patient journeys. In the third stage, SRF extracted data from all 22 studies included in this review [20, 21]. The variables extracted in this second stage involved components of patient journeys, including journey terminology, format of depiction, and starting and ending points. Additional variables extracted included: notes on treatment and diagnosis; whether or not referrals were a part of the pathway and if so, the rationale for referral; care delivery providers, including community health workers (CHWs), government or Ministry of Health clinics, traditional healers, private clinics, or non-governmental organisations (NGOs), and barriers and facilitators of care continuity. The final data dictionary of 46 total variables is available in S3 text. One researcher (SRF) extracted full texts in both stages. At the end of the third stage, a second researcher (EO) verified extraction of a random 10% of included studies, resulting in the final extraction table (S1 Dataset). Synthesis of patient journeys was conducted by categorising NTDs into groupings based on having similar disease presentations, approaches to care management, or aetiologies. For each NTD grouping, framework synthesis was used to identify and link key components of the patient journeys in the included literature [22, 23]. The result of this synthesis was a simplified, consolidated schematic for each NTD or NTD grouping which depicts possible journeys NTD patients may undertake while accessing care over time and given the progression of disease, accompanied by a narrative description of the patient journey, based on the available patient journey literature at the time of this review.

## Results

**Fig. 1:**
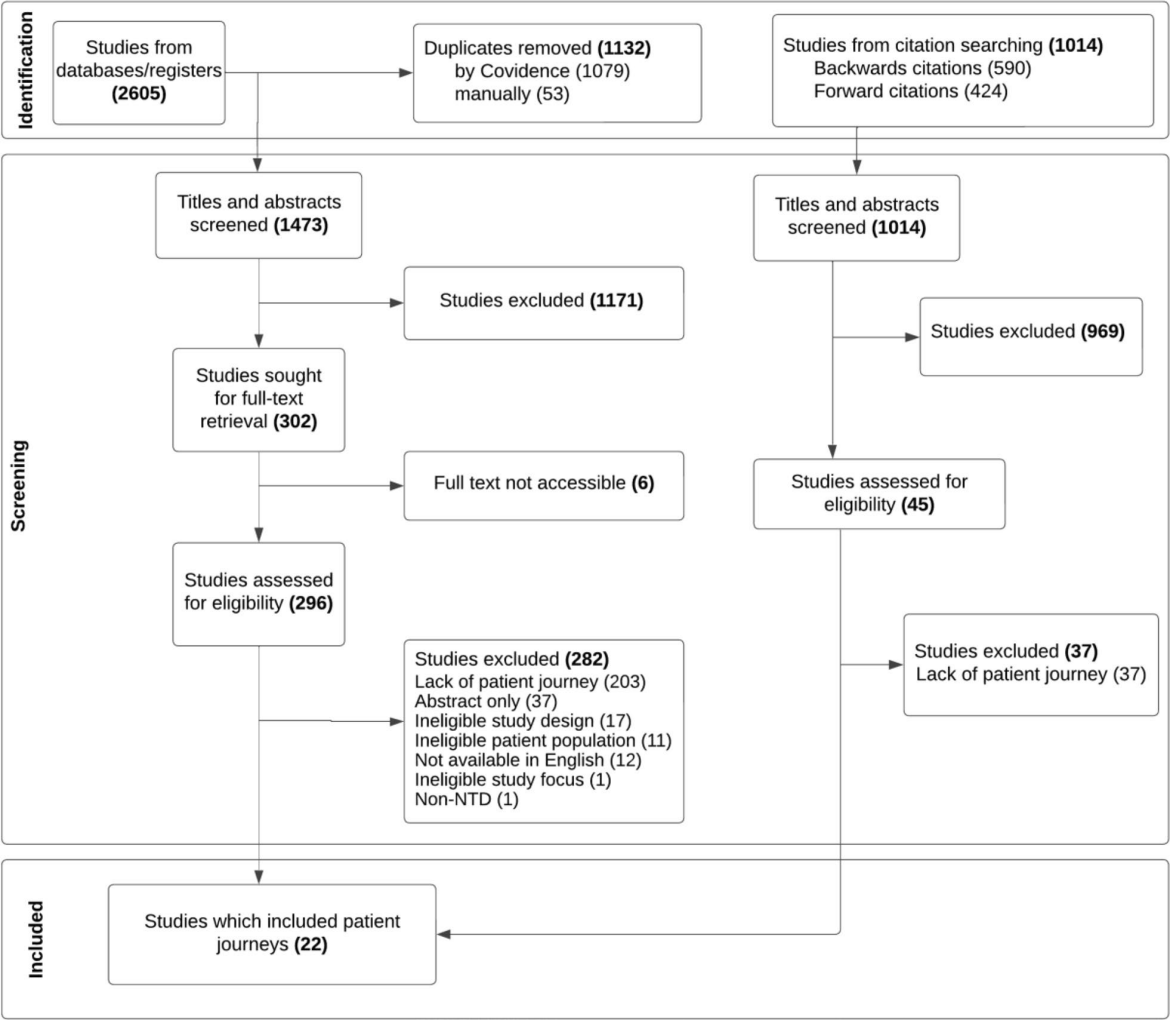
Preferred Reporting Items for Systematic reviews and Meta-Analyses extension for Scoping Reviews (PRISMA-ScR) study selection flowchart. The screening and review process is split into three stages: 1) the identification of articles through an initial search, 2) the screening of articles following predefined inclusion and exclusion criteria and 3) the final included articles for data extraction

The initial database search was run in February 2023 and resulted in 2178 studies. Following deduplication, 1257 titles and abstracts were screened and 963 were deemed ineligible, leaving 294 studies. Of these, 11 studies were identified for inclusion. The second running of the search in November 2024 identified an additional 427 studies, 211 of which were duplicates. The remaining 216 studies were screened at title and abstract level, with eight screened at full text level. Of these, three met the inclusion criteria. Through backward and forward citation searching of these 14 studies identified through the database search, we identified 45 studies for full text screening. Of these, two studies identified through backward citation searching and six studies identified through forward citation searching met the inclusion criteria, culminating in 22 total studies qualifying for inclusion (S4 text). Figure 1 details the search process for this systematic scoping review and the combined number of studies identified, screened, and excluded from both runs of the systematic search and citation screening.

We identified 22 eligible studies with patient journeys from 1696 individual NTD patients or patient caregivers. Summary characteristics of the journeys presented in these studies are shown in Table 2. In these patient journeys, eight different NTDs were represented: HAT, Buruli ulcer, lymphatic filariasis, onchocerciasis, schistosomiasis, yaws, snakebite, and rabies. Patient journeys were primarily depicted through detailed narratives (72.1%; 16/22), though patient journeys were also shown in a range of visual formats including flowcharts and maps. The average number of NTD patient participants was 77 (Standard deviation 97), with sample sizes for each study ranging from 1 to 96 patients, and 54.5% (12/22) of studies involving 30 participants or less [4–6, 24–32]. Studies which engaged more participants used multiple focus group discussions or shorter interviews to develop a synthesised narrative, while smaller studies used in-depth or life-history interviews to understand individual experiences**.** Just over half (55.4%; 12/22) of studies were on NTDs requiring innovative and intensified diseases management (HAT, Buruli ulcer, and yaws) [3–6, 24–26, 29, 32–35], and 18.2% (4/22) of studies were on NTDs caused by bite injury requiring emergency response (snakebite and rabies) [27, 36–38]. Nineteen studies (86.4%) focused on only one NTD, while three studies (13.6%) focused on multiple NTDs, all of which were SSSDs [25, 31, 39]. NTDs for which MDA of preventative chemotherapy (PC) is the core strategic intervention (schistosomiasis, onchocerciasis, lymphatic filariasis) were represented in five studies [28, 30, 31, 39, 40], though MDA of PC is also used as an intervention component for two conditions in three studies on multiple SSSDs (leprosy and yaws) [25, 31, 39].

**Table 2:** Summary table of characteristics of included studies

Author and year	Aim	Country	Setting	Study design	NTD(s)	Pathway Name	Pathway representation	Pathway start	Pathway end
Ackumey 2012 [33]	To examine socio-cultural determinants of timely medical treatment for pre-ulcers and delayed medical treatment for ulcers in an endemic area in Ghana	Ghana	Mixed	Semi-structured interviews	Buruli ulcer	Illness narratives/help-seeking behaviour	Narrative	Onset of infection	Unspecified, with difficulty adhering to treatment regimes
Anyolitho 2023 [40]	To assess (1) the communities’ sources of health care services for the treatment of schistosomiasis-related signs and symptoms and, (2) the factors determining their choices of health-seeking regarding schistosomiasis-related signs and symptoms in the absence of the mass drug administration program, to inform government and partners on how to improve praziquantel drug uptake	Uganda	Community	In-depth interviews, focus group discussions	Schistosomiasis	Modified conceptual framework of health-seeking behaviour	Flowchart	Onset of symptoms	Receiving treatment, or pursuit of urgent care
Aujoulat 2003 [24]	To better understand what people know, think and feel about Buruli ulcer and treatment facilities; to see which cognitive and emotional obstacles prevent patients from presenting to the health care centre; to identify terms used locally to describe Buruli ulcer in order to incorporate them into health information campaign messages; and to determine the most appropriate people for delivering such health education messages	Benin	Community	Focus group discussions	Buruli ulcer	Therapeutic itinerary	Narrative	Cause of disease	Unspecified; scars, amputation, or fear of recurrence
Barrett 2024 [30]	To consider (1) the lived experiences of the syndemic and current healthcare response; and (2) the impact of an enhanced self-care intervention on the syndemic relationship, with a view to make recommendations to improve the mental and physical wellbeing of people affected by Lymphatic Filariasis in Malawi	Malawi	Community	Life history interviews	Lymphatic filariasis	Life-history narratives/stories	Narrative	Onset of symptoms	Improvement following enhanced self-care intervention
Bukachi 2009 [5]	To document the treatment-seeking pathways followed by human African trypanosomiasis cases in the areas of Western Kenya and eastern Uganda that together form the Busoga Focus	Multiple/across borders—Uganda, Kenya	Mixed	Interviews	Human African Trypanosomiasis	Treatment pathways	Narrative + flowchart of misdiagnoses	Onset of symptoms	Diagnosis, and subsequent start of human African trypanosomiasis treatment
Dean 2019 [31]	To use feminist intersectional theory as a key analytical lens to consider how individuals’ unique positions of power and privilege and can shape their experiences and to reflect on what this means for the creation of responsive, people centred health systems in Liberia	Liberia	Mixed	Interviews	Leprosy, Buruli ulcer, lymphatic filariasis, onchocerciasis	Illness narratives/health seeking pathways	Narrative	Onset of symptoms	No clear ending; “freedom” from physical disease though emotional traumas remained; participant goals to return to life circumstances before the onset of illness
Kibadi 2009 [6]	To describe lay perceptions of the ulcerated forms of Mycobacterium ulcerans, commonly called Buruli Ulcer, and therapeutic itineraries of Buruli Ulcer patients in a rural area of the DRC	Democratic Republic of the Congo	Facility	Interviews	Buruli ulcer	Therapeutic itineraries	Narrative + flowchart	Onset of symptoms	Specific treatment at health centre
Koka 2020 [32]	To determine health seeking behaviour for Buruli ulcer by affected persons and their families. Specifically, the study was designed to examine the reasons why Buruli ulcer patients and their families seek treatment from home, traditional healers and the biomedical health facilities	Ghana	Community	In-depth interviews	Buruli ulcer	Health seeking behaviour	Narrative	Onset of symptoms	Treatment at a health facility
Kone 2020 [4]	To describe the health seeking pathway of a girl who was the first human African trypanosomiasis patient diagnosed through the national strategy for passive surveillance of human African trypanosomiasis as integrated into the national health system and based on clinical suspicion	Cote d’Ivoire	Facility	Epidemiological and clinical investigation	Human African trypanosomiasis	Health seeking pathway/health-seeking itinerary	Narrative + map of geographical itinerary	Onset of symptoms	Diagnosis (parasitological)
Larson 2022 [37]	To characterize snakebite incidence, risk, factors, and subsequent health seeking behaviours in two regions of Kenya using a mixed methods approach	Kenya	Community	Interviews	Snakebite	Incident narratives (bite incident narratives)	Narrative	Exposure (snakebite)	Long term outcomes
Masong 2024 [28]	To explore challenges that emerge as a result of cultural constructions around gender and structural gaps in the provision of care, stemming from individual perceptions and community views of a person affected as a result of their manifestation of female genital schistosomiasis symptoms	Cameroon	Community	In-depth interviews	Schistosomiasis (female genital)	Illness narratives	Narrative	Participation in FGS diagnostic activities	Initiation of treatment; long term mental distress
McCollum 2024 [39]	To draw on mixed-methods approaches to further contextualise the syndemic relationship between NTDs (and their associated disability) and mental distress (depression, anxiety) in Liberia	Liberia	Community	In-depth interviews, participatory methods (body mapping and social mapping)	Leprosy, Buruli ulcer, lymphatic filariasis, onchocerciasis	Care seeking pathways	Narrative	Diagnosis	Unspecified
Menlah 2020 [29]	To identify the psychological, socioeconomic, and coping strategies encountered by the victims with Buruli ulcer in Amasaman	Ghana	Community	In-depth interviews	Buruli ulcer	Steps taken after diagnosis	Narrative	Onset of symptoms	Discharge from hospital for home treatment; possibility of full recovery
Mulder 2008 [34]	To describe the steps that patients with Buruli ulcer go through before reporting to the hospital/treatment centre	Benin	Community	Semi-structured interviews	Buruli ulcer	Model for healthcare seeking behaviour	Flowchart	Onset of infection	Multiple endings; either healed or active disease, either at home or through hospital treatment
N’Guessan 2022 [38]	To describe and analyse the social determinants of non-adherence to post-exposure prophylaxis treatment that occurred during the period of free health care in San Pedro	Cote d’Ivoire	Mixed	Observation, interviews, focus group discussions	Rabies (animal bite injury)	Therapeutic itineraries	Narrative	Exposure; rabies vaccination centre	End of treatment, either full series or early drop out
Ogechi 2022 [26]	To identify the characteristics of Buruli ulcer-infected households, their health-seeking behaviour that influences how they perceive the disease, and the results of care for Buruli ulcer disease in Owerri, Imo state	Nigeria	Community	In-depth interviews	Buruli ulcer	Health seeking behaviours	Narrative	Onset of symptoms	Leaving treatment centre upon not noticing a change in their health; scarring or amputation
Okyere 2024 [25]	To investigate the local health system’s readiness to provide care; perceptions and experiences of skin NTDs and services by people affected, their families, and the wider community, and perspectives on policy and practice challenges of key actors at district, regional, and national levels	Ghana	Mixed	In-depth interviews, focus group discussions	Buruli ulcer, yaws, leprosy	Care seeking pathways/Care-seeking journeys	Flowchart	Onset of symptoms	No clear ending; wound still healing or almost healed
Palmer 2014 [3]	To evaluate the results of a syndromic human African trypanosomiasis training intervention which targeted healthcare workers in peripheral facilities with the objective of increasing the rate of syndromic referrals to a central screening and treatment centre	South Sudan	Mixed	Interviews, focus group discussions	Human African trypanosomiasis	Treatment seeking and test referral pathways	Flowchart	Infection	Screening and diagnostic tests conducted
Peeters Grietens 2012 [35]	To assess the role beliefs play in determining Burulil ulcer sufferers’ choice between traditional and biomedical treatments	Cameroon	Mixed	Observation, interviews, focus group discussions	Buruli ulcer	Therapeutic itineraries/health seeking itineraries/health itineraries	Narrative + table	Care seeking	Unspecified
Schurer 2022 [36]	To describe human activities and environment immediately preceding snakebite and characterise experiences accessing formal and informal care	Rwanda	Community	In-depth interviews	Snakebite	Care seeking routes	List	Care seeking	Arrival at hospital or traditional healer
van Oirschot 2021[27]	To gain in-depth insights into the views and experiences of Kenyan community members with regard to snakebite prevention and care	Kenya	Community	Focus group discussions	Snakebite	Health seeking pathways	Narrative	Exposure (snakebite)	Visiting a health facility
Weg 1998 [41]	To study explanatory models and help seeking behaviour of leprosy patients	Nigeria	Facility	Interviews	Leprosy	Delay and help-seeking flow diagram	Flowchart	Onset of symptoms	Accessing leprosy services

*NTDs* neglected tropical diseases

The patient journey components identified included: disease origins; infection; onset of symptoms; decision to seek care; care seeking; diagnosis, referrals, treatment; and long-term outcomes. Additional components underpinning the patient journey framework included disease progression over time and the local context of healthcare access. While 50% (11/22) of studies began patient journeys at the onset of symptoms [4–6, 25, 26, 29–32, 40, 41], the journeys in the other seven studies indicated varying starting points ranging from exposure, to onset of infection, to care seeking. As indicated in Table 1, none of the studied journeys ended exact the same way. Journey endings varied from arrival at the health facility, to diagnostic tests conducted, to diagnosis confirmed, to start or end of treatment, to lack of ending given perpetuation of chronic disease. Only 36.4% (8/22) of these studies continued the journey beyond the start of treatment [24, 29, 30, 33, 34, 37–39]. Of these, experiences beyond the start of treatment reflected difficulties with treatment adherence or fear of disease recurrence. All 22 included studies mentioned government or Ministry of Health clinics as care providers. Twenty one studies (95.5%) also mentioned use of traditional healers, though one study on rabies described use of traditional healers among less than 1% of NTD patients [38]. Under half of all studies (9/22) also referenced use of private healthcare providers or drug shops [3–5, 25, 29, 30, 32, 36, 40], while 9.1% (2/22) also mentioned a non-governmental or aid organisation in care provision [26, 35]. One study (7.1%) was described as embedded within a larger research study on patient self-care [30]. CHWs were referenced as actors providing care management in 32% (7/22) of studies describing patient journeys [3, 4, 27, 31, 36, 38–40]. More than half of all studies (55.4%; 12/22) referenced referrals as part of the patient journey [3, 5, 25, 27, 31, 33, 34, 37–40]. Reasons for referral included lack of treatment availability locally [27, 36], lack of diagnostic capacity or need for a confirmatory diagnosis [3, 5, 34], or upon treatment failure [31, 36].

### Distinctions between patient journeys by NTD

To synthesise patient journeys, NTDs were grouped based on having similar disease presentations, approaches to care management, or aetiologies. These groupings included SSSDs, HAT, rabies and snakebite [14], and schistosomiasis (intestinal and female genital) [13].

### SSSDs

**Fig. 2:**
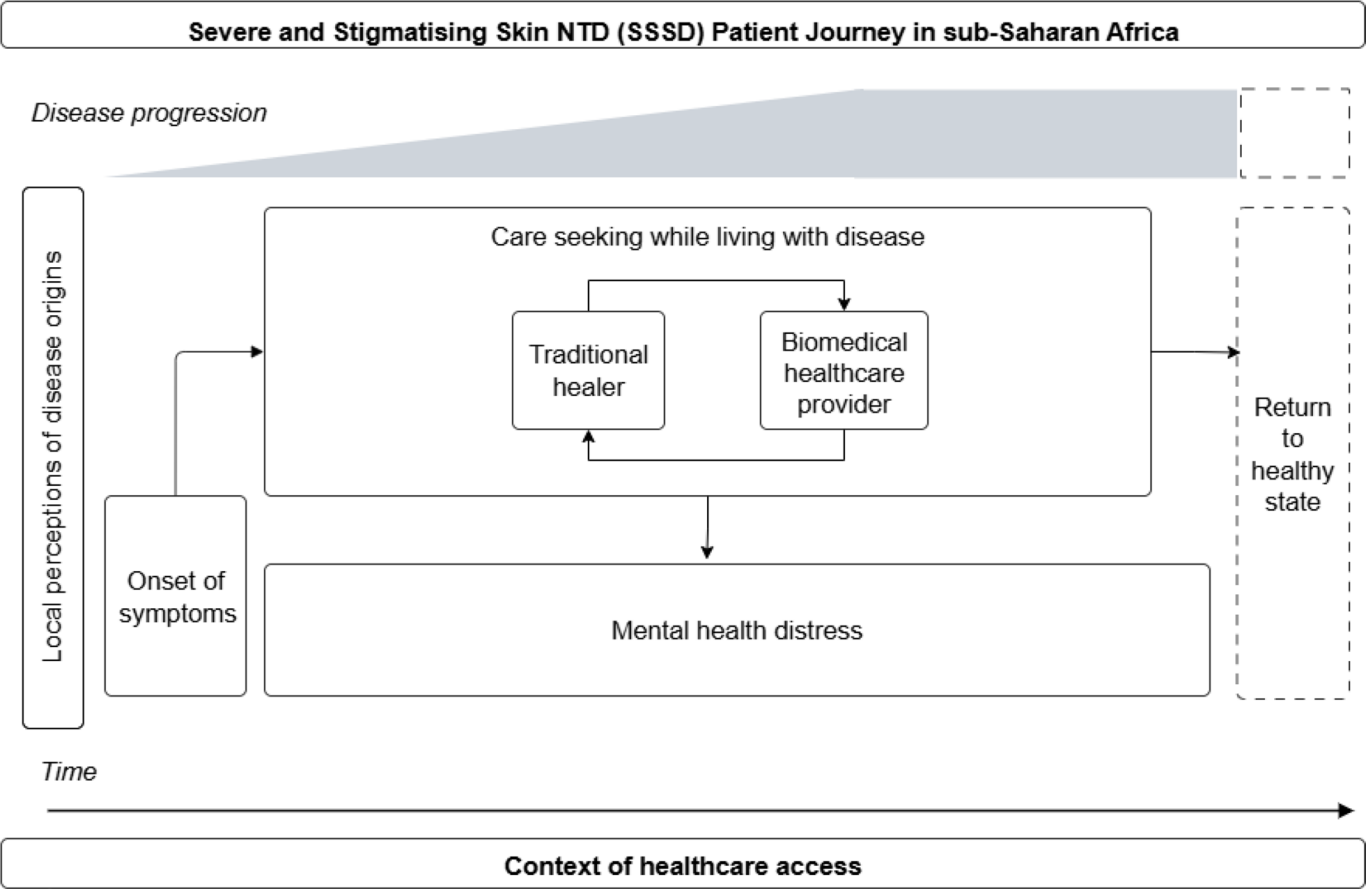
Simplified severe and stigmatising skin NTD (SSSD) patient journey in sub-Saharan Africa. This schematic depicts possible journeys SSSD patients may undertake while accessing care over time and given the progression of disease from the perspective of the patient. This schematic draws from self-reported data to highlight patient actions and decision making, which may differ from clinically recommended treatment algorithms. This schematic is not intended to represent all patient journeys or possible outcomes, and is limited to the available patient journey literature for SSSDs

SSSDs including Buruli ulcer, leprosy, lymphatic filariasis, and onchociasis were the focus of the majority of studies (59%; 13/22). Of these, 11 of included patient experiences with chronic Buruli ulcer [6, 24–26, 29, 31–35, 39], while one focused on lymphatic filariasis [30], and one focused on leprosy [41]. Three studies described patient journeys for individuals living with either Buruli ulcer, leprosy, lymphatic filariasis, or onchocerciasis as conditions with similar early clinical presentations yet different disease aetiologies and a range of required management approaches [25, 31, 39]. SSSD patient journeys minimised infection as a journey component and emphasized the challenges of long-term treatment and chronic management, particularly with regard to patient mental health. A simplified visual representation of an SSSD patient journey as represented in the literature is available in Fig. 2.

Patient journeys for SSSDs were grounded in local, spiritual perceptions of disease origins [31, 34, 35]. However, SSSDs were also understood to have biomedical disease dimensions, with all studies describing consultation and treatment required by both traditional healers and biomedical healthcare providers for comprehensive disease management. Importantly, SSSD patient journeys were described as non-linear, where patients often sought symptom management prior to receiving a diagnosis, and obtaining a diagnosis did not necessarily result in treatment [34]. To support disease identification early in the patient journey, sensitization was recommended for clinicians and traditional healers, as well as the general public, given the role neighbours or community members can play in recommending further care [6, 34]. Obtaining SSSD diagnoses was reported as complicated, either due to insufficient diagnostic capacity at locally accessible health centres [6], or because of initial non-specific disease presentations [29, 35]. Among patients who did receive a diagnosis, many did not want to accept their diagnosis given the lack of efficacious options available for treatment, resulting in repeated re-consultation with healthcare providers and continued treatment attempts with traditional healers [31, 34, 35]. For those who did accept their diagnosis, there was a sense of anxiety regarding the uncertainty of possible healing [39].

SSSD patient journeys entailed ongoing challenges accessing care given the disabling effects of disease. These challenges primarily involved stigma, mental health distress, and the absence of a clear referral pathway or effective treatment regimen [25, 30, 31, 34, 35, 39]. During the later stages of the patient journey, multiple studies emphasized the mental health challenges experienced by SSSD patients given the protracted trauma of living with a disabling, isolating, and stigmatising condition with no straightforward treatment in a resource-constrained setting [25, 30, 31, 39]. One study explicitly mentioning the lack of respect patients received from medical providers and local health facilities due to their condition [35]. Two of the studies on multiple SSSDs, both based in Liberia, highlighted the role of context as an important factor in traumatic patient experiences [31, 39]. One of these studies on multiple NTDs in Liberia recommended the involvement in SSSD patients in patient-specific support groups to address psychosocial challenges experienced by SSSD patients following decades of civil unrest which resulted in fragmented local healthcare infrastructure [31]. The study on lymphatic filariasis, which was embedded within a larger study on enhanced self-care, indicated potential for improved patient wellbeing and outcomes for patients engaged in enhanced self-care practices [30]. The enhanced self-care activities in this study entailed WHO-recommended wound care practices alongside changes in diet and activities to cultivate mindfulness [30].

While the return to a healthy pre-disease state was described as possible in two studies [29, 34], five studies culminated in accessing treatment [6, 29, 32, 34, 41]. Two studies concluded with either scarring or amputation, or anxiety about these possible treatment outcomes [24, 26]. Five studies presented inconclusive endings given the continued experience of chronic disease and the need for long term case management, as prolonged infection may not necessarily lead to death, but rather a plateau of severe physical disability that may be aggravated by mental distress [25, 31, 33, 35, 39].

### HAT

**Fig. 3:**
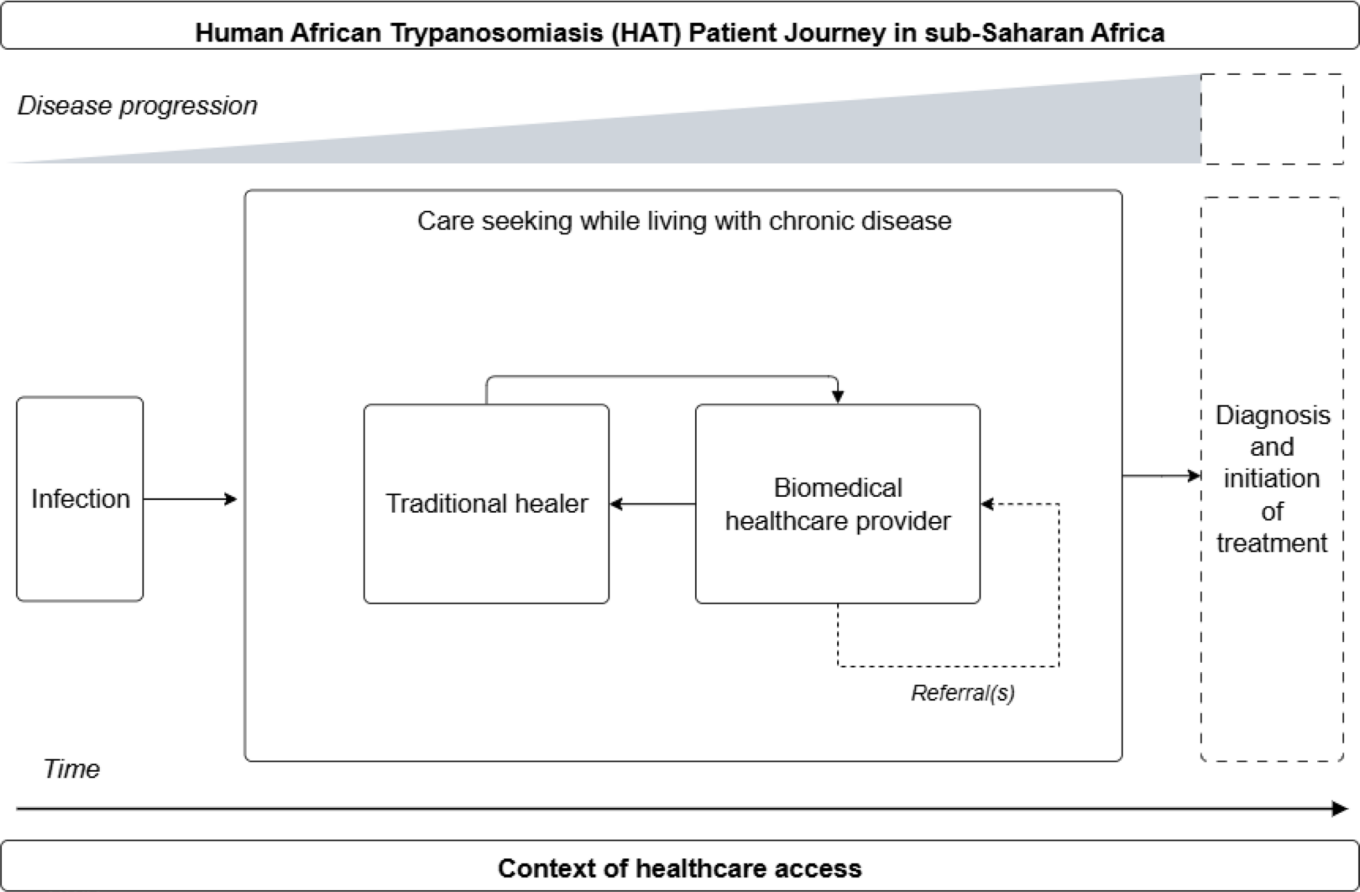
Simplified human African trypanosomiasis (HAT) patient journey in sub-Saharan Africa. This schematic depicts possible journeys HAT patients may undertake while accessing care over time and given the progression of disease from the perspective of the patient. This schematic draws from self-reported data to highlight patient actions and decision making, which may differ from clinically recommended treatment algorithms. This schematic is not intended to represent all patient journeys or possible outcomes, and is limited to the available patient journey literature for HAT

HAT was the disease of focus in 13.6% (3/22) of patient journeys identified in this review. A simplified visual representation of a HAT patient journey as represented in the literature is available in Fig. 3.

While HAT patient journeys began with either onset of symptoms or infection, HAT patient journeys emphasized the prolonged process of reaching diagnosis, with misdiagnosis as a key journey component. Several reasons for frequent misdiagnosis were presented. In HAT-endemic settings of Cote d’Ivoire, South Sudan, Uganda, and Kenya where HAT patient journey literature was represented, HAT diagnosis and treatment was limited to dedicated, centralized HAT testing and treatment centres accessed by referral, with inadequate diagnostic and treatment capacity elsewhere [3–5]. In this context, healthcare providers outside of these dedicated centres were often unaware of HAT as a possible diagnosis. Multiple reasons were given for why healthcare providers lacked HAT awareness. These reasons included lack of HAT sensitization for local healthcare workers, perceptions of HAT as a disease from the past century, and stigma associated with HAT due to disease associations with recent civil conflict and mistaken concerns of sexual transmission [3–5]. Additionally, frequent false positives and discordant results between HAT screening and parasitological diagnostic testing created overwhelming complexity for healthcare workers, leading to disinterest and disengagement with disease identification and management [3, 4].

Due to the complexity of diagnosis-seeking in the patient journey and the non-specific nature of early disease presentation, HAT patients typically engaged in symptom management or attempted malaria treatment before receiving a confirmatory diagnosis [3]. Referrals to further testing were provided only if symptoms did not improve following malaria treatment [3]. Given the non-specific presentations, suspect patients often received joint referrals for HAT, malaria, and typhoid testing. Two studies emphasized the importance of increased awareness among healthcare workers for prompt HAT referral and diagnosis [4, 5]. However, the third study noted how this may increase referrals for suspect cases to confirmatory diagnosis and treatment, but increased sensitization will not address the structural challenges rooted in poverty that hinder referral completion [3]. For many patients, transportation to centralized referral centres was a significant obstacle in the patient journey [3]. While some patients used personal funds to access referrals, others relied on advocacy from local healthcare workers to opportunistically coordinate transport, such as through military vehicles, local non-governmental organisations, or shared use of ambulances [3]. All HAT patient journeys ended with diagnosis and possible start of treatment. No study provided detailed information on HAT patient journeys following treatment initiation.

### Snakebite and rabies

**Fig. 4:**
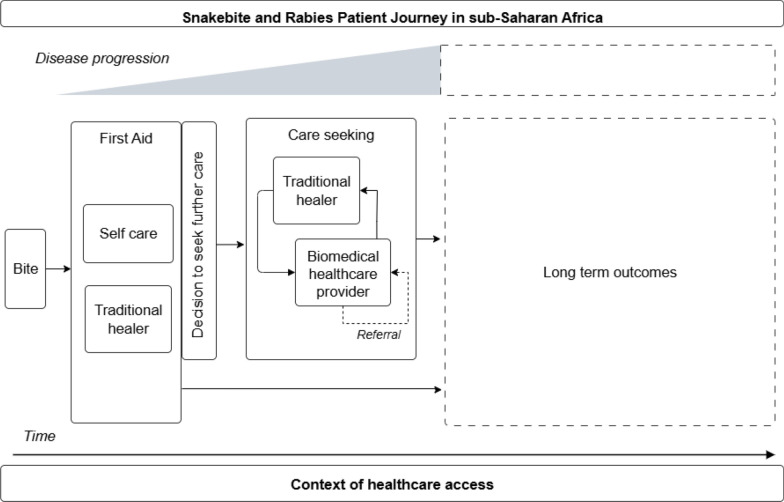
Simplified snakebite and rabies patient journey in sub-Saharan Africa. This schematic depicts possible journeys a snakebite or rabies patient may undertake while accessing care over time and given the progression of disease from the perspective of the patient. This schematic draws from self-reported data to highlight patient actions and decision making, which may differ from clinically recommended treatment algorithms. This schematic is not intended to represent all patient journeys or possible outcomes, and is limited to the available patient journey literature for snakebite and rabies

Snakebite and rabies were the focus of 18.2% (4/22) patient journeys identified in this review [27, 36–38]. These patient journeys emphasized obstacles in accessing emergency response once the decision was made to access care. A simplified visual representation of a patient journey for snakebite and rabies as represented in the literature is available in Fig. 4.

Three studies showed patient journeys beginning with the snake or animal bite as the point of exposure [27, 37, 38]. Immediately following exposure, most individuals bitten by a snake or potentially exposed to rabies typically sought first aid using traditional methods, or self-administered wound care [36, 38]. However, few individuals potentially exposed to rabies accessed biomedical health facilities immediately following first aid despite the importance of post-exposure prophylaxis. The vast majority of potentially rabies-exposed individuals waited to access further care depending on the worsening of symptoms, and were unaware that free pre-exposure prophylactic vaccination was available at a local vaccination centre [38]. For individuals bitten by a snake, the push to access further care was more urgent than potential rabies exposure given community awareness of snakebite as a health issue and a priority [37]. For both snakebite and animal bite patients, the patient journey involved back and forth between multiple provider types based on treatment failure, referral between providers, or loss of trust [36].

Inaccessible care was described as an issue in snakebite and potential rabies patient journeys due to lack of antivenom at local health facilities and insurmountable distance of either primary or referral health facilities from patient sites [27, 36, 37]. For snakebite patients these challenges were connected, as lack of antivenom was a reason for referral or facility transfer, and delays in referrals can have severe, life-limiting consequences [27, 37]. To address challenges with snakebite referrals, two studies recommended the provision of antivenom at local health facilities and placement of a trained doctor in the village or training in first aid at the community level, alongside increased sensitisation of community members and traditional healers, and improved infrastructure [27, 36]. Another study described the utility of local community health workers having access to phones and SMS services to support snakebite patient referral and follow up [36]. To improve referrals for individuals potentially exposed to rabies, further training of healthcare workers to improve local vaccination uptake was encouraged [38].

In two studies, the patient journey ended at contact with either a traditional or biomedical provider [27, 36]. The remaining two studies explored long term outcomes. For snakebite, long term outcomes ranged from chronic pain and discomfort, to long term hospital admissions ranging from several weeks to multiple years, to risk of death [37]. In the study on rabies, the journey ended at either conclusion of treatment, defined as either completion of the full treatment series or non-completion [38]. Reasons for non-completion of the rabies post-exposure prophylaxis series included insufficient information regarding the necessity of post-exposure prophylaxis, distance to health facilities, and ineffective procedures for follow-up [38].

### Schistosomiasis

**Fig. 5:**
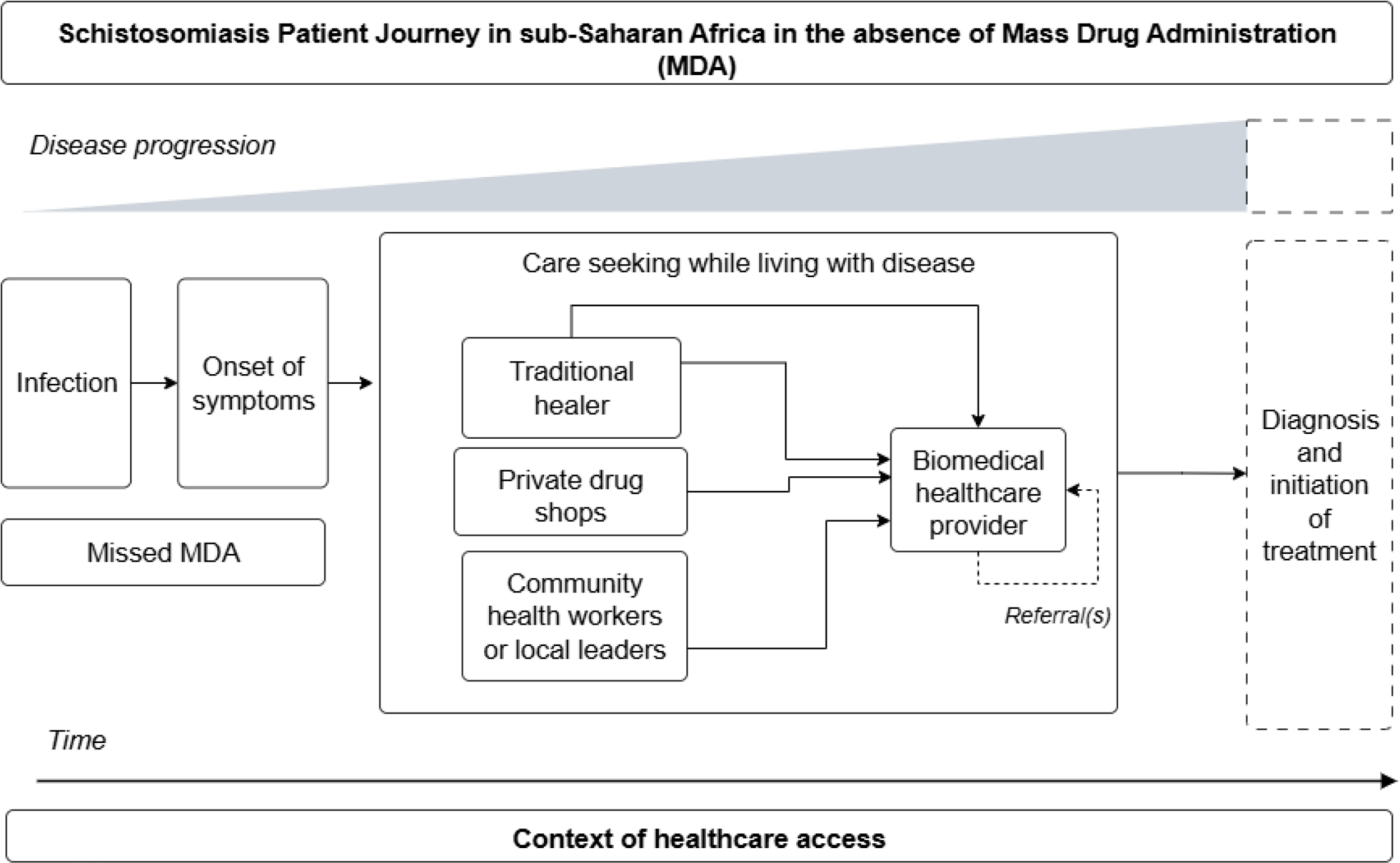
Simplified schistosomiasis patient journey in sub-Saharan Africa. This schematic depicts possible journeys a schistosomiasis patient may undertake while accessing care over time and given the progression of disease from the perspective of the patient. This schematic draws from self-reported data to highlight patient actions and decision making, which may differ from clinically recommended treatment algorithms. This schematic is not intended to represent all patient journeys or possible outcomes, and is limited to the available patient journey literature for schistosomiasis

Schistosomiasis was the focus of 9.1% (2/22) of patient journeys identified in this review [28, 40]. Female genital schistosomiasis (FGS) (*Schistosoma haematobium*) and intestinal schistosomiasis (*S. mansoni*) were examined in separate studies. A simplified visual representation of the patient journey for schistosomiasis as represented in the literature is available in Fig. 5.

Schistosomiasis patient journeys emphasized care seeking in the context of missed MDA of praziquantel as PC, regardless of the species of schistosome and associated diverse clinical outcomes. For the study on intestinal schistosomiasis, MDA was considered missed because it did not take place within the MDA timeframe of at least every 12 months in highly endemic areas, as recommended by the WHO [40, 42]. For the study on FGS, MDA activities were described as periodically ongoing in the study setting. However, MDA was still missed by FGS patients due to gendered dynamics of household decision making where women were not allowed to participate without the approval from their husband, the distance of campaigns from patient villages, and campaigns which exclusively targeted school children as opposed to general populations at high-risk of exposure [28].

In the absence of MDA, the challenges individuals faced in accessing care for schistosomiasis treatment and management were extensive. As depicted in the patient journey literature for schistosomiasis, in Uganda and Cameroon FGS or intestinal schistosomiasis diagnosis was not typically available through local health centres due to a lack of diagnostic tools [28, 40]. For individuals who were able to access diagnostics, or for individuals choosing to forgo diagnosis and seek treatment directly based on their experience of early-stage schistosomiasis symptoms, accessing praziquantel for treatment presented substantial difficulties. While some individuals were successful accessing praziquantel through local leaders or community health workers left to distribute the surplus following MDA activities, or through local drug shops, structural barriers complicated access to further treatment. Structural barriers included health system and infrastructural constraints, such as lack of accessible roads, distance from health facilities, high financial costs associated with treatment, and lack of trained medical personnel [40]. In this context, some individuals attempted treatment through traditional healers who were selected not necessarily out of preference but due to their accessibility and low cost [40]. In both studies, social dynamics of gender hindered a woman’s agency to seek treatment, and perceived stigma from healthcare workers towards schistosomiasis patients deterred patients from seeking care [28, 40]. For FGS patients, development of severe disease was associated with mental health distress, particularly given the impact of the disease on fertility [28]. Upon progression of intestinal schistosomiasis into severe disease and life-threatening complications, such as vomiting blood due to bursting of oesophageal varices, one study mentioned that patients would pursue urgent care at biomedical health facilities [40].

To improve treatment accessibility and care continuity in schistosomiasis patient journeys, it was recommended that praziquantel not only be accessible through MDA, but be consistently available in local health centres in endemic communities [40]. While the study on intestinal schistosomiasis described referrals to specialized facilities for management of severe disease [40], the end of the schistosomiasis patient journey for both intestinal schistosomiasis and FGS entailed taking praziquantel as initiation of treatment or pursuit of urgent care. Neither study provided detailed information on schistosomiasis patient journeys following treatment initiation for either early stage or advanced disease.

## Discussion

Through this scoping review, we identified 22 studies which included patient journeys from 1696 individuals affected by eight different NTDs in SSA. These patient journeys were depicted through flow diagrams, lists of ordered journey components, or patient narratives. Within these patient journeys, we identified the following journey components: disease origins; infection; onset of symptoms; decision to seek care; care seeking; diagnosis, referrals, treatment; and long-term outcomes. These components did not necessarily appear in a linear order, at times overlapped, and were emphasized or de-emphasized depending on the corresponding journey characteristics. Based on the patient journey literature, we synthesised visual patient journeys and produced narrative patient journey summaries for four groupings of NTDs: Buruli ulcer, leprosy, lymphatic filariasis, and onchocerciasis as SSSDs; HAT; snakebite and rabies; and schistosomiasis. This stratification provides a robust foundation for understanding both common and disease-specific bottlenecks in care pathways, allowing for comparison of patient journeys between NTDs groupings and illuminating evolving experiences of healthcare access for NTD patients.

The conceptualisation of patient journeys presented in this review broadly aligns with therapeutic itineraries as a framework for understanding patient-directed narratives of care seeking in LMIC settings where health systems infrastructure may be limited, with origins in Brazilian medical anthropology in the 1990s [43]. Through narratives, diagrams, and schematics, patient journeys enable understanding of patient experiences over time by illuminating the full arc of care seeking throughout multiple phases of disease progression. As a journey, the patient journey is inherently progressive, implying a non-static experience of engaging with multiple possible healthcare providers in an attempt to seek care over time. In the context of NTDs, time is crucial, as it underpins both the urgency of providing care for emergent NTDs such as snakebite, and the protracted, progressively debilitating nature of NTDs known to have chronic and disabling affects. This framing of progressive care needs over time is helpful for understanding patient experiences with NTDs that have been documented through scientific research as requiring chronic care, such as Buruli ulcer and other SSSDs that comprise the majority of studies included in this review. Additionally, the extended lens of the long-term patient journey aids in understanding NTDs for which scientific evidence of chronic morbidity and its management may be limited, such as schistosomiasis, HAT, rabies, and snakebite.

NTDs occur in environments characterised by systemic neglect and health inequity [44]. This shared context of extreme poverty shapes NTD patient experiences of healthcare access in resource-constrained settings and underpins all NTD patient journeys in this scoping review. Patient journeys for different NTDs highlight specific dimensions of this context. For snakebite, the lack of antivenom at local health centres and difficulty completing urgent referrals exposed challenges relating to supply chain constraints for antivenom as a medical commodity alongside structural and financial barriers to referral and transport for emergency response [27, 36–38]. For HAT as an NTD with largely non-specific symptoms and a centralized case management structure typically accessed through referrals, patient journeys emphasized limited accessibility of local diagnostics and issues with referrals for advanced care [3–5]. For schistosomiasis as an NTD which relies on MDA as a primary intervention, patient journeys underscored how disease-specific delivery models shape NTD patient experiences [40]. As shown in this review, vertical treatment models such as that of schistosomiasis can limit access to care when preventive treatment is not available outside of MDA implementation [40]. The FGS patient journey stressed gendered dynamics that constrain female agency and restrict healthcare seeking [28]. Each of these different challenges—accessibility of medical resources and diagnostics, limitations of existing referral systems, reliance on MDA, and gendered social structures—reflect important aspects of the substantial financial, geographic, social, and structural barriers to NTD care as highlighted by McCollum et al. [10].

Within this local context of healthcare access, we identified a lack of disease awareness, social and perceived stigma, roles of traditional healers, and referrals as prominent features of NTD patient journeys. Half of all NTD patient journeys in this review, including all HAT and snakebite patient journeys, highlighted a lack of awareness in NTD identification, management, and referral among healthcare providers or community members [3–5, 27, 31, 34, 37]. However, experiences from HAT patient journeys describe how addressing lack of awareness through increased sensitization is not sufficient for enabling care continuity, as sensitization alone does not address the structural challenges rooted in poverty that hinder referral completion [3]. For SSSDs, HAT and FGS, lack of NTD awareness was intertwined with stigma and corresponding mental health distress. Stigma experienced by HAT and SSSD patients took different forms, including the stigmatization of SSSD disability [25, 30, 31, 39], and stigma towards HAT patients from clinicians who viewed the disease as overly complex to diagnose and manage [4]. For women affected by FGS, stigma intersected with gender norms and limited treatment-seeking due to anticipated judgment from healthcare workers [28]. Based on this review, in contexts where biomedical services were inaccessible due to financial constraints or stigma, traditional healers often serve as the first and most accessible point of contact. The majority of studies referenced referrals as part of the patient journey, for reasons extending from inability to provide diagnosis or treatment locally, or treatment failure [3, 5, 25, 27, 31, 33, 34, 37–40]. In NTD contexts, these aspects of NTD journeys underscore the need for integrated NTD care models that bolster awareness and address stigma while engaging traditional healers and strengthening referral pathways to care through local health systems.

Distinctions between patient journeys were salient in the differences between journey endings—or lack of endings, for each NTD grouping. In five of the 13 studies on SSSDs, the ending of the patient journey was undefined, suggesting that for SSSDs, the experience of living with long term pain and disability does not necessarily have a conclusion, even after accessing treatment [25, 31, 33, 35, 39]. For both studies on schistosomiasis, the patient journey ended at access to praziquantel, thereby emphasizing treatment access and the lack of alternatives such as morbidity case management [28, 40]. For snakebite and rabies, two of four included studies culminated in accessing a biomedical health facility [27, 36], emphasizing the importance of prompt biomedical care. These endings reflect a risk of mortality without treatment, in contrast with SSSDs. However, the remaining studies on snakebite and rabies focused on long term outcomes [37, 38], highlighting how snakebite and rabies may require both emergency and long-term care. In the case of rabies in particular, N’Guessan et al. highlight that the patient journey concludes at the end of treatment, whether that is completion of a full series of post-rabies exposure prophylaxis, or early drop-out from treatment [38]. That treatment completion is not an essential component of the rabies patient journey emphasizes the likelihood that treatment may not be completed and the corresponding difficulty continued treatment may present over an extended time period, despite the clinical importance of post-rabies exposure prophylaxis for patient outcomes. In the case of HAT, patient journeys culminated in seeking diagnosis and possibly initiating treatment. However, HAT patient journeys did not incorporate patient experiences of care continuity during long term treatment, despite how advanced HAT may require several months of treatment and up to 2 years of clinical follow up [3–5, 11]. While patient journey endings diverged in meaningful ways depending on disease characteristics and treatment modalities, the current literature on NTD patient journeys shows an emphasis on initial care seeking as opposed to care continuity, and a lack of emphasis on care for long-term or chronic conditions.

The WHO Roadmap for Neglected Tropical Diseases 2021–2030 recommends that NTD interventions include cross cutting, multi-sectoral approaches that are not limited to a single preventive or management strategy [19]. However, the patient journeys in this review indicated a lack of integrated approaches for NTD prevention, treatment, and management. In particular, this was shown through limited studies [3] on patient journeys and morbidity management for onchocerciasis and schistosomiasis as NTDs that have MDA as the core intervention. In the case of schistosomiasis as indicated in this review, MDA may be considered missed if it does not take place within the WHO recommended timeframe [40, 42]. When MDA for schistosomiasis does take place, implementation is imperfect and can miss or exclude high risk populations [45, 46], thereby undermining MDA goals of reduction of transmission and prevention of disease. For intestinal schistosomiasis, chronic infection due to missed MDA in schistosomiasis-endemic areas can result in nonspecific gastrointestinal symptoms, which may develop into severe gut and liver morbidities requiring urgent and complex care [47]. While Anyolitho et al. describe care seeking and referrals for severe intestinal schistosomiasis morbidities [40], no further information was provided on what this treatment entails or whether it was accessible. This lack of further treatment is reflected in the lack of clinical care guidelines for managing the morbidities resulting from chronic schistosomiasis infection within low-income health systems [48]. To develop care guidelines that extend beyond MDA and include morbidity management and clear referral pathways, there is a need for further research on patient journeys for schistosomiasis.

As a framework and in the context of this review, patient journeys have several limitations. Each patient journey is the result of a unique and complex set of factors and decisions. While the synthesized journeys in this review highlight at the types of experiences and challenges NTD patients may have while seeking care as represented in the literature, actual NTD patient journeys extend beyond the literature to the lived experiences of NTD patients. Relatedly, the synthesized patient journeys in this review are limited to the available patient journey literature and may not necessarily include the entirety of the patient experience in relation to a particular NTD. This is clear in the absence of long-term treatment from HAT patient journeys and the omission of management of severe intestinal morbidities from schistosomiasis patient journeys. Given the fluid nature of terminology relating to patient journeys, patient journeys may be interpreted differently by different researchers, which may result in different points of emphasis.

Beyond framework selection, this review has a few additional limitations. The limited number of studies which met the inclusion criteria may limit generalisability, particularly for NTDs which were only represented in one or two studies. Several NTDs endemic in SSA, such as soil-transmitted helminthiases, are entirely not represented in the included literature. As a scoping review, this study did not assess evidence quality or risk of bias in included articles. Additionally, the English language restriction may have excluded publications from francophone and lusophone SSA or reports in local languages, leading to possible underrepresentation of patient journeys from countries where English is not a primary language.

## Conclusions

Patient journeys illuminate the evolving experience of healthcare access for NTD patients over the course of time and disease progression. While this study frames NTD care through the people-centred and structurally aware lens of patient journeys, its limited disease representation and focus on acute care interactions point to the need for more granular, longitudinal and community-embedded approaches to understand and intervene in NTD patient care seeking experiences. The synthesised NTD patient journeys in this review show a range of care seeking experiences within the broader context of neglect and health inequity that characterises settings where NTDs are endemic. By understanding the journeys NTD patients take when seeking care and how these challenge manifest through social, structural, and health systems constraints rooted in the local context, NTD researchers and practitioners can determine how best to support NTD patients in navigating access to care for acute and chronic conditions.

## Supplementary Information

Supplementary Material 1. Prisma ScR Checklist. Checklist of PRISMA reporting criteria for scoping reviews.

Supplementary Material 2. Search string and database hits. Full search string and table of database hits.

Supplementary Material 3. Data dictionary. Table of 46 variables for extraction.

Supplementary Material 4. References included. List of 22 studies qualifying for inclusion.

Supplementary Material 5. Dataset NTDPJ. Complete data extraction table of 22 included studies and 46 variables.

## Data Availability

All data generated or analysed during this study are included in this published article (S1 Dataset).

## Abbreviations

CHWs: Community health workers
FGS: Female genital schistosomiasis
HAT: Human African trypanosomiasis
LMICs: Low- and middle-income countries
MDA: Mass Drug Administration
NTDs: Neglected tropical diseases
PC: Preventative chemotherapy
PRISMA-ScR: Preferred Reporting Items for Systematic Reviews and Meta Analyses extension for Scoping Reviews
SSA: Sub-Saharan Africa
SSSDs: Severe and stigmatising skin NTDs
WHO: World Health Organization
