# Supplementary material for: Patient journeys for neglected tropical diseases in rural sub-Saharan Africa: a scoping review
Source: Infect Dis Poverty. 2025 Nov 6;14:112. doi: 10.1186/s40249-025-01385-7 (PMC12590602; doi:10.1186/s40249-025-01385-7)
Supplement: Supplementary file 3 — Supplementary Material 3. Data dictionary. Table of 46 variables for extraction. [file 40249_2025_1385_MOESM3_ESM.docx]

**S3 Text: Data Dictionary**

|  | **Variable** | **Definition** | **Proposed options** |
| --- | --- | --- | --- |
| 1 | **author_year** | Last name of first author | [Name year] |
| 2 | **Search result** | Through which search was the study identified? | February 2023, November 2025, Backward citation screening, Forward citation screening (Google Scholar) |
| 3 | **Title** | Full title | [Title] |
| 4 | **DOI** | DOI or link | [DOI or link] |
| 5 | **Abstract** | Abstract | [Abstract] |
| 6 | **publication_year** | Year of publication | [Year] |
| 7 | **country** | In which country did the study take place? | Sub-Saharan Africa |
| 8 | **country_region** | In what region within the study country? | [Describe] |
| 9 | **study_setting** | What was the study setting? | Community, Facility, Mixed |
| 10 | **study_aims** | What were the research aims(s) of the study? | [Describe] |
| 11 | **study_design** | What was the design of the study? | Prospective cohort, retrospective cohort, case study, case-control, before and after study, cross-sectional, comparative cross-sectional, repeat cross-sectional, Randomized Controlled Trial |
| 12 | **study_data_collection** | What were the qualitative methodologies used to eliciting data from NTD patients? | Survey, interviews, focus group discussion, etc. |
| 13 | **study_design_data_type** | Did the study only use qualitative data, or did it complement the qualitative data with mixed methods? | Qualitative; Mixed methods |
| 14 | **study_data_collection_other** | If the study used any other methods, either to complement qualitative methods or for non-patient participants, what were they? | Survey, clinical observation, key informant interviews, hospital data |
| 15 | **study_intervention** | If the study was a randomised controlled trial or involved an evaluation of an intervention, what was the intervention? | [Describe] |
| 16 | **study_comparison** | If the study included an intervention what was the control group/comparison? | [Describe] |
| 17 | **study_disease_main** | What was the primary disease being investigated in the study? | All NTDs |
| 18 | **study_disease_secondary** | What was the secondary disease being investigated in the study? | All NTDs |
| 19 | **study_disease_other** | Were other NTD(s) being investigated in the study? | All NTDs |
| 20 | **study_disease_other_2** | Were other NTD(s) being investigated in the study? | All NTDs |
| 21 | **study_condition_non_NTD** | Was the disease being managed alongside any other health conditions/comorbidites (not NTDs)? | All diseases and health conditions, (excluding NTDs) |
| 22 | **sample_size** | What is the number of patients (current, former, or suspect) in study sample who participated in qualitative data collection? | Total number of participants |
| 23 | **age_groups** | What were the age categories of the study participants? | Children, Adults, Children and Adults/All |
| 24 | **study_participant_patients** | Were current (suspect or confirmed) or former patients participants in the study? Participants in a screening campaign/prevalence study count as suspect patients. | Suspect patients, Current patients, former patients, guardians if underage |
| 25 | **study_participants_other** | Aside from NTD patients, were there any other participants in the study? | I.e. Family members or guardians of current or former patients, Community Health Workers, Clinicians, Key Informants (NGO workers, Ministry of Health Staff), etc |
| 26 | **care_delivery_providers_gov** | Did NTD patients seek or attempt to seek care from government/MOH health facilities? | Yes, No |
| 27 | **care_delivery_providers_traditional** | Did NTD patients seek or attempt to seek care from traditional healers? | Yes, No |
| 28 | **care_delivery_providers_other** | Did NTD patients seek or attempt to seek care from any other facilities or healthcare providers? | I.e. private clinics, drug shops, NGOs |
| 29 | **chws_yn** | Were Community Health Workers mentioned in the study? | Yes, No |
| 30 | **chws_roles_care_yn** | If Community Health Workers were involved in the study, did they play a role in patient care? | Yes, No, Unspecified, N/A |
| 31 | **chws_roles_care** | If Community Health Workers were involved in the study and played a role in patient care, what was that role? | Delivery of medications, follow up on referrals, participation in health promotion, etc |
| 32 | **patient_pathways_yn** | Did the study explicitly describe a sequence of events related to diagnosis, treatment, and follow up from the patient perspective (the patient pathway)? | [Describe] |
| 33 | **patient_pathways_terminology** | If "yes" to variable patient_pathways_yn, what was the terminology used to describe the sequence of events related to diagnosis, treatment, and follow up from the patient perspective (the patient pathway)? | [Describe] |
| 34 | **patient_pathways_depiction** | If "yes" to variable patient_pathways_yn, how as the patient pathway depicted? | [Describe] |
| 35 | **patient_pathways_start** | If "yes" to variable patient_pathways_yn, what was considered the start of the pathway? | [Describe] |
| 36 | **patient_pathways_end** | If "yes" to variable patient_pathways_yn, what was considered the end of the pathway? | [Describe] |
| 37 | **patient_pathways_single** | If "yes" to variable patient_pathways_yn, was there one primary patient pathway presented, either through focus on an individual case study, or through synthesis? | Yes, No |
| 38 | **patient_pathways_multiple** | If "yes" to variable patient_pathways_yn, were multiple possible patient pathways presented and/or were the pathways non-linear (i.e. multiple attempts seeking diagnostics from different providers prior to treatment)? | Yes, No |
| 39 | **patient_pathway_notes** | Any other details regarding the patient pathway and/or healthcare seeking in health system | [Describe] |
| 40 | **diagnosis_misdiagnosis** | Did the study explicitly reference misdiagnosis, treatment for another disease, or discordant results? | Yes, No |
| 41 | **diagnosis_notes** | Any other information regarding the diagnosis/suspect case assessment? | [Describe] |
| 42 | **treatment_ notes** | Any other information regarding the treatment, including any additional levels of treatment or why specific treatment options were chosen? | [Explain] |
| 43 | **referrals_yn** | Did the study explicitly mention referrals? | No, Yes, Yes but only as part of ethics statement |
| 44 | **referrals_why** | If the study explicitly mentioned referrals, why was the referral was given? i.e. Why the referral was necessary or why care could not be provided locally | Treatment following prevalence study, Treatment not available locally or immediately, Suspect case in need of diagnostic confirmation |
| 45 | **referral_notes** | Any other notes regarding the referral? | [Describe] |
| 46 | **study_other** | Any other notes regarding the study? | [Describe] |
