# Supplementary material for: Patient journeys for neglected tropical diseases in rural sub-Saharan Africa: a scoping review
Source: Infect Dis Poverty. 2025 Nov 6;14:112. doi: 10.1186/s40249-025-01385-7 (PMC12590602; doi:10.1186/s40249-025-01385-7)
Supplement: Supplementary file 4 — Supplementary Material 4. References included. List of 22 studies qualifying for inclusion. [file 40249_2025_1385_MOESM4_ESM.docx]

# S4_References included

1. Ackumey MM, Gyapong M, Pappoe M, Maclean CK, Weiss MG. Socio-cultural determinants of timely and delayed treatment of Buruli ulcer: implications for disease control. Infect Dis Poverty. 2012 Oct 25;1(1):6.

2. Anyolitho MK, Nyakato VN, Huyse T, Poels K, Masquillier C. Health-seeking behaviour regarding schistosomiasis treatment in the absence of a mass drug administration (MDA) program: the case of endemic communities along Lake Albert in Western Uganda. BMC Public Health. 2023 June 5;23(1):1072.

3. Aujoulat I, Johnson C, Zinsou C, Guedenon A, Portaels F. Psychosocial aspects of health seeking behaviours of patients with Buruli ulcer in southern Benin. Tropical medicine & international health : TM & IH. 2003;8(8):750–9.

4. Barrett C, Chiphwanya J, Matipula DE, Douglass J, Kelly-Hope LA, Dean L. Addressing the Syndemic Relationship between Lymphatic Filariasis and Mental Distress in Malawi: The Potential of Enhanced Self-Care. Tropical Medicine and Infectious Disease. 2024 Aug;9(8):172.

5. Bukachi S.A., Wandibba S., Nyamongo I.K. The treatment pathways followed by cases of human African trypanosomiasis in western Kenya and Eastern Uganda. Annals of Tropical Medicine and Parasitology. 2009;103(3):211–20.

6. Dean L, Tolhurst R, Nallo G, Kollie K, Bettee A, Theobald S. Neglected tropical disease as a “biographical disruption”: Listening to the narratives of affected persons to develop integrated people centred care in Liberia. PLoS neglected tropical diseases. 2019;13(9):e0007710.

7. Kibadi K., Boelaert M., Kayinua M., Minuku J.-B., Muyembe-Tamfum J.-J., Portaels F., et al. Therapeutic itineraries of patients with ulcerated forms of Mycobacterium ulcerans (Buruli ulcer) disease in a rural health zone in the democratic Republic of Congo. Tropical Medicine and International Health. 2009;14(9):1110–6.

8. Koka E. Health seeking behaviour for Buruli Ulcer disease in the Obom sub-district of the Ga south Municipality of Ghana. International Journal of Public Health. 2020 Mar 21;4:18.

9. Kone M, N’Gouan EK, Kaba D, Koffi M, Kouakou L, N’Dri L, et al. The complex health seeking pathway of a human African trypanosomiasis patient in Cote d’Ivoire underlines the need of setting up passive surveillance systems. PLoS neglected tropical diseases. 2020;14(9):e0008588.

10. Larson PS, Ndemwa M, Thomas AF, Tamari N, Diela P, Changoma M, et al. Snakebite victim profiles and treatment-seeking behaviors in two regions of Kenya: results from a health demographic surveillance system. Tropical medicine and health. 2022;50(1):31.

11. Masong MC, Mengue MT, Marlene NT, Dean L, Thomson R, Stothard R, et al. Illness experiences and mental health challenges associated with female genital schistosomiasis in Cameroon: a gender analysis. International Health. 2024 Apr 1;16(Supplement_1):i42–51.

12. McCollum R, Barrett C, Zawolo G, Johnstone R, Godwin-Akpan TG, Berrian H, et al. ‘The Lost Peace’: Evidencing the Syndemic Relationship between Neglected Tropical Diseases and Mental Distress in Liberia. Tropical Medicine and Infectious Disease. 2024 Aug;9(8):183.

13. Menlah A, Appiah EO, Boahemaa MK. Experiences of Buruli Ulcer Patients Following Discharge in the Greater Accra Region of Ghana. The International Journal of Lower Extremity Wounds. 2022 Sept 1;21(3):303–11.

14. Mulder AA, Boerma RP, Barogui Y, Zinsou C, Johnson RC, Gbovi J, et al. Healthcare seeking behaviour for Buruli ulcer in Benin: a model to capture therapy choice of patients and healthy community members. Transactions of the Royal Society of Tropical Medicine and Hygiene. 2008;102(9):912–20.

15. N’Guessan R.D., Heitz-Tokpa K., Amalaman D.M., Tetchi S.M., Kallo V., Ndjoug Ndour A.P., et al. Determinants of Rabies Post-exposure Prophylaxis Drop-Out in the Region of San-Pedro, Cote d’Ivoire. Frontiers in Veterinary Science. 2022;9((N’Guessan, Amalaman) Sociology Department, Universite Peleforo Gon Coulibaly, Korhogo, Cote D’Ivoire(N’Guessan, Heitz-Tokpa, Bonfoh) Centre Suisse de Recherches Scientifiques en Cote d’Ivoire, Abidjan, Cote D’Ivoire(Tetchi) Institut National d’Hygiene Pu):878886.

16. Ogechi N, Chigozie Divine O, Ekeanyanwu C. Household Perceptions, Treatment Seeking Behaviour, and Health Outcomes for Buruli Ulcer Disease in Owerri, South-Eastern Nigeria. American Journal of Nursing Science. 2022 Sept 21;11:123–33.

17. Okyere D, Ocloo EK, Owusu L, Amoako YA, Tuwor RD, Koka E, et al. Improving experiences of neglected tropical diseases of the skin: Mixed methods formative research for development of a complex intervention in Atwima Mponua District, Ghana. SHARP collaboration, editor. PLOS Glob Public Health. 2024;4(6):e0002833.

18. Palmer JJ, Surur EI, Checchi F, Ahmad F, Ackom FK, Whitty CJM. A mixed methods study of a health worker training intervention to increase syndromic referral for gambiense human African trypanosomiasis in South Sudan. PLoS neglected tropical diseases. 2014;8(3):e2742.

19. Peeters Grietens K, Toomer E, Um Boock A, Hausmann-Muela S, Peeters H, Kanobana K, et al. What role do traditional beliefs play in treatment seeking and delay for Buruli ulcer disease?--insights from a mixed methods study in Cameroon. PloS one. 2012;7(5):e36954.

20. Schurer JM, Dam A, Mutuyimana MT, Runanira DM, Nduwayezu R, Amuguni JH. “At the hospital they do not treat venom from snakebites”: A qualitative assessment of health seeking perspectives and experiences among snakebite victims in Rwanda. Toxicon: X. 2022;14(101741983):100100.

21. van Oirschot J, Ooms GI, Okemo DJ, Waldmann B, Reed T. An exploratory focus group study on experiences with snakebites: health-seeking behaviour and challenges in rural communities of Kenya. Transactions of the Royal Society of Tropical Medicine and Hygiene. 2021;115(6):613–8.

22. Weg NVD, Post EB, Lucassen R, Jong JTVMD, Broek JVD. Explanatory models and help-seeking behaviour of leprosy patients in Adamawa State, Nigeria. Leprosy Review [Internet]. 1998 [cited 2025 Aug 16];69(4). Available from: https://www.academia.edu/53109700/Explanatory_models_and_help_seeking_behaviour_of_leprosy_patients_in_Adamawa_State_Nigeria
